# The Influence of Likes and Sexist Attitudes on Adolescent Self-Esteem in Social Networks

**DOI:** 10.3390/ijerph21121647

**Published:** 2024-12-10

**Authors:** Yéxica Flores Valdés, Antonio Daniel García-Rojas, Angel Hernando Gómez, Javier del Rio Olvera

**Affiliations:** 1 Department of Social, Developmental and Educational Psychology, University of Huelva, 21071 Huelva, Spain; jexica.flores@dpee.uhu.es (Y.F.V.); angel.hernando@dpsi.uhu.es (A.H.G.); 2 Department of Pedagogy, University of Huelva, 21071 Huelva, Spain; 3 Department of Psychology, Institute for Biomedical Research and Innovation of Cadiz (INiBICA), University of Cádiz, 11510 Puerto Real, Spain; franciscojavier.delrio@uca.es

**Keywords:** social networks, self-esteem, sexism, adolescents, identity

## Abstract

The Internet allows teenagers to express their identity through the publication of images and texts on social networks, but sometimes they may develop self-esteem problems as a result. The present study analyzed self-esteem levels, and their relationship with sexism, Internet use and the influence of likes, in 309 subjects, by asking them about Internet use, social networks, self-esteem and sexism. The results showed low levels of self-esteem, although boys scored higher on the overall scale and for hostile sexism. Similarly, those with higher percentages of low self-esteem showed higher scores for benevolent sexism. It can be concluded that the use of social networks and the Internet, in relation to sexist attitudes, influences adolescents’ self-esteem and social construction.

## 1. Introduction

In recent years, with the advent of the Internet, a technological revolution has taken place that has promoted the development and massification of tools that enable a real and global exchange of information [1,2]. We live in an era in which everything is interconnected, and the Internet is an added space that makes it possible to overcome the limitations of space–time to a greater extent. New contexts are constantly being generated to express and explore aspects of identity, and information and communication technologies (ICTs) are an optimal channel for approaching and understanding young people, who make great use of these tools, incorporating them in their daily lives, in their communications and connections [1]. Among these new contexts, the sites most frequented by adolescents are social networks, followed by video sharing platforms [3]. Some of these social networks have a direct influence on the construction of identity, and adolescents can link with their peers on platforms such as Facebook and YouTube [4] or Tik Tok, Instagram and Snapchat; these last three, according to the evidence, are considered to be highly visual social networks, since almost exclusively visual content is shared [5]. Recently, they have become the most popular networks, leaving behind traditional ones, such as Facebook or messaging applications. Moreover, their use is fully embedded in adolescents’ daily lives, with studies indicating that in addition to entertainment and the search for information, social networks are used as a means of interaction with those they consider to be their peers [2].

Social networks allow adolescents to satisfy emotional, relational or social needs, and to communicate with their peers [2,6]. Adolescents use them to contact each other, exchange information or share their emotions, and receiving likes or positive comments is a sign of popularity and acceptance [6]. This promotes social integration and satisfies the need for belonging and affiliation by making visible and reaffirming their identity [5,6]. On the contrary, not receiving likes or positive comments can have negative consequences on emotional and psychological well-being by causing adolescents to feel excluded or ignored [6]. The importance of teens receiving likes could be related to the FOMO phenomenon, or the fear of missing out. This is the need and well-being that comes from being aware of what others are doing while maintaining online connection and interactions [7]. This implies a belief of connection with others that promotes psychological well-being and satisfies the need to belong to the group; however, it increases the risk of excessive use of social networks and worsening of adolescent self-esteem [8]. The satisfaction of these needs that social networks allow directly influences the exposure that takes place on the Internet, in such a way that the more satisfaction adolescents receive, the more content they will share on social networks, and the greater sense of belonging they will perceive [6]. Therefore, it is essential to develop adaptive strategies in the use of social networks, since they have a great impact on the health and social adaptation of adolescents, which are central aspects of youth identity [9].

Nowadays, the way to approach and understand the online life of young people is, in general, through an image that the subject uses as a letter of introduction in the virtual medium, and that can be assumed to have an intentionality prior to its exposure [10], since adolescents use the publication of images and texts to build their identity, which creates significant body and virtual image issues [10,11,12]. This translates into adolescents experiencing excessive concern about their physical appearance, and an overvaluation of the body in both sexes, especially in terms of negative Body Image (CI) [13]. An example of this is musculature, which is traditionally associated with masculinity and power in men and is currently gaining importance among adolescent girls, and seems to have a significant influence on body image [7]. In this sense, social networks and body image play a very important role in acceptance by a group, since the former can make adolescents think that a positive body image facilitates social interactions and increases belonging [14]. Thus, negative body image influences interpersonal relationships [7,15].

The use of photography on social networks has become widespread and normalized, and both boys and girls post images on their profiles without knowing the consequences, sometimes becoming reckless in what they post and the content that they express [13,16], which can lead to risky online behaviors that compromise the safety of adolescents, such as sexting or expanding their contact list [17]. According to evidence, adolescent girls who experience lower levels of body self-esteem, or negative body image, are more likely to engage in unsafe online behaviors, such as expanding their social network indiscriminately and developing intimate relationships with strangers they meet online [17]. According to the literature, one explanation for these behaviors could be the aim of obtaining positive feedback from other people, which, related to self-esteem, explains, to a certain extent, online behavior and the publication of virtual images, in that subjects with a higher self-esteem share photos with many more users [17,18].

These publications affect gender socialization, leading to stereotypes associated with boys and girls, and carrying an associated sexist bias that cannot be traced to a reference figure or to education received [19]. These sexist attitudes and values that are maintained through ICTs explain the perpetuation of gender inequalities in adolescents, which seems to be influenced by sociocultural patterns, and leads to the maintenance of sexist constructs [20]. The “Ambivalent Sexism Theory” [21], which includes both hostile or traditional sexism, defined as an attitude of prejudice or discriminatory behavior based on the supposed inferiority or difference of women as a group, and benevolent sexism, which is understood as a set of interrelated attitudes towards women that are sexist in that they are stereotyped and limited to certain roles, but have a positive affective tone (for the recipient), and tend to elicit behaviors typically categorized as prosocial (helping) or intimacy-seeking (self-disclosure). Some studies show that, in general, men have higher levels of hostile sexism, but women tend to have similar or higher scores on benevolent sexism [22].

Considering all the above, and according to the literature, negative peer appraisal in adolescence is one of the factors that causes body dissatisfaction disorders to develop early on, leading to emotional problems. This is even more relevant and complicated in the present day, as socialization occurs mainly through ICTs and social networks. Therefore, it is especially relevant to promote the formation of a subjective identity and a social life that leads to equal relationships free of sexist attitudes and, as a later consequence, violence. Thus, the objectives of the present study on the positive influence of likes on teenagers in social networks are as follows: (i) to explore the levels of self-perceived personal self-esteem in adolescents; (ii) to determine the relationship between self-esteem and sexist attitudes; (iii) to analyze the influence of likes on adolescents’ self-esteem; and, finally, (iv) to study the time and type of Internet use by adolescents, and the reasons and motivations for their Internet use.

## 2. Methodology

### 2.1. Participants

The study sample consisted of a total of 309 participants, secondary school students from four state-run secondary schools in the province of Huelva, who were accessed through convenience sampling and in person. Of the total, 51.5%, i.e., 158 participants, were girls, compared to 48.5% (148) boys, with a mean age of 15 years (SD = 0.58).

The sample inclusion criteria were (a) being over 13 years old and under 17 years old; (b) having daily access to Internet and social networks; and (c) students in the 3rd and 4th years of secondary education, to ensure a better understanding and validity of the tests applied. The exclusion criteria were not obtaining parental consent, not having access to social networks, or being under 13 years old or over 17 years old.

### 2.2. Instruments

An ad hoc questionnaire was used, designed by the research team and composed of a total of 34 items. Items were included to collect some personal data, such as sex and age, and modules were incorporated to find out what reactions the subjects had to the appearance of a peer or, alternatively, to their absence. The questionnaire was in paper format and designed to collect relevant information for the study. The administration time to complete the survey was approximately 15 min.

The version validated in Spanish [21] of the Ambivalent Sexism Inventory for Adolescents [21] was used. It consists of 22 items theoretically grouped into two subscales: hostile sexism (11 items) and benevolent sexism (11 items). The items present a response format with 6 alternatives, ranging from 1 point (strongly disagree) to 6 points (strongly agree). It provides a measure of hostile sexism and a measure of benevolent sexism, calculated from the mean of the scores obtained in each of the items of the subscales. The authors of the validated Spanish version reported a reliability of 0.910. In the present research, a Cronbach’s alpha of 0.901 was reached.

The Rosenberg Self-Esteem Scale [23], originally developed for the assessment of self-esteem in adolescents, was used. The scale includes ten items whose contents focus on feelings of self-respect and self-acceptance. Half of the items are positively stated, and half are negatively stated. The responses use a four-point Likert-type scale (1 = strongly agree, 2 = agree, 3 = disagree, 4 = strongly disagree). For their correction, the scores of the items stated negatively (3, 5, 8, 9, 10) should be inverted, and then all the items should be added together. The total score, therefore, ranges between 10 and 40. In the Spanish adaptation [24], the alpha coefficient is 0.880. In the present research, a Cronbach’s alpha of 0.850 was reached.

### 2.3. Procedure

Seven secondary schools were selected in the province of Huelva, where adolescents who met the requirements of the research were available. The research team then contacted the head teacher or, failing that, the guidance counselor of the school to explain the research objectives, as well as to ask for their collaboration. Finally, four of the seven schools agreed. The students were asked to cooperate, and it was always ensured that all information collected was confidential, anonymous and voluntary. Through the school, the participants and their parents/legal guardians were asked to sign a written informed consent form describing the nature and purpose of the study. In addition, school principals gave their consent for students to participate in the study.

Participants were asked to fill in the questionnaires on paper with a pen. The questionnaires were administered directly by the authors and psychologist collaborators trained in the classroom, after a date and time agreed with the tutor of the degree. It was announced that participation was voluntary and that there was no reward. The participants were informed that they could withdraw from the questionnaire at any point without repercussions, and that participation did not imply any harm. Data gathering took place between March and April 2023.

The self-esteem variables were categorized into two differentiating categories: low self-esteem and medium-high self-esteem. The sexism variable was also categorized into two categories: hostile sexism and benevolent sexism.

## 3. Analysis and Results

1. Self-perceived personal self-esteem in adolescents

Two self-esteem groups were established, categorized into low and medium-high self-esteem. In total, 88% of the students scored for low self-esteem and 12% for medium-high self-esteem. Regarding the differences by sex, boys scored 44.7 and girls scored 43.4 for low self-esteem. For medium-high self-esteem, boys scored 6.8 and girls scored 5.2. These results were not statistically significant.

2. Relationship between self-esteem and sexist attitudes

Boys (50.9%) scored higher for benevolent sexism than girls (49.1%). Regarding hostile sexism, boys also scored higher, with 50.8%, so the results did not show statistically significant differences. Those with higher percentages of low self-esteem showed higher scores for benevolent sexism, with 88.2%. Similar scores were found for hostile sexism, with 88.1% for low self-esteem.

3. Influence of likes on adolescent satisfaction and self-esteem

52.8% claimed that they were not influenced by likes on social networks; however, 25.2% expected to receive many likes, and only 0.3% expected few likes. For those who did not receive many likes, higher percentages reported that they did not care and it did not bother them (77.7%), although 15.2% reported that they did not care, but it did bother them. On the other hand, for those who did receive a lot of likes, 53.4% did not care, 18.1% continued to upload content with the same frequency, and 15.5% felt good and loved on the networks.

71.5% said that the number of likes did not influence their satisfaction and personal well-being, and 17.2% reported that the number of likes affected them.

4. Time, type of use, and reasons and motivations for Internet use

First, the descriptive data of the questionnaire items are presented. The number of respondents answering Yes, No or Not sure is indicated. Table 1 shows the frequency and percentage for each.

Subsequently, among those who answered Yes to a question, we checked whether there were significant differences between low scores below the 25th percentile and high scores above the 75th percentile for self-esteem, benevolent sexism and hostile sexism. These results are shown in Table 2. To decide the type of contrast to be performed to verify the study hypotheses, the Kolmogorov–Smirnov test (Lilliefors correction) was carried out to check the assumption of normality of the data. It was found that both the self-esteem questionnaire (sig. ≤ 0.000) and the data on benevolent sexism (sig. ≤ 0.000) and hostile sexism (sig. ≤ 0.000) did not meet the normality assumption. For this reason, we decided to use non-parametric contrasts.

Regarding the time spent connected to the internet, 32.4% connected between 10 and 20 h a week, and only 17.2% connected for 40 h or more. The most used social networks were WhatsApp (97.4%), Instagram (94.8%) and YouTube (90.9%).

## 4. Discussion

Categorizing self-esteem into low and medium-high, the results show higher scores in both self-esteem groups for boys than for girls; however, these differences are not statistically significant [25]. When comparing self-esteem scores by gender, it is boys who score higher than girls for self-esteem [26]. According to the literature, it seems that body image problems also affect boys, but to a lesser extent than girls [27]. The lower the self-esteem, the greater the magnitude of body image problems in adolescents, and the lower their academic performance and unproductive behaviors in some areas of the social sphere [25,27]. However, although special attention should be paid to this group, the levels of low self-esteem should not be alarming, as self-esteem is most affected in the adolescent stage. Even so, more in-depth study of the factors that influence self-esteem during adolescence is relevant in order to understand how to enhance adolescents’ abilities and self-concept, since these are bolstered as they enter adulthood. This is consistent with the outcomes of the present study, in which we observed that older adolescents had lower scores for low self-esteem, which could suggest that as we move through the life cycle and enter adulthood, self-esteem is strengthened.

Although the results do not show significant differences in sexism between boys and girls, there is a slight variation in hostile sexism, for which boys scored higher. Therefore, there seems to be a relationship between hostile sexism and negative stereotypes about women in the case of boys, and benevolent sexism and positive stereotypes in both sexes. Of relevance is the relationship between benevolent sexism and lower self-esteem in girls, as shown by the following results.

These sexist stereotypes are reinforced by various television phenomena, as well as by advertising, which reinforce social attitudes such as gender in colors or diets, influencing the social construction of the gender stereotypes of girls. Sexist attitudes that eroticize the submissiveness of women and enhance the aggressiveness and dominance of men are explicitly reinforced [22,28]. Social networks have become the perfect vehicle for disseminating these sexist stereotypes that influence the self-esteem of both boys and girls.

There was a group of students who reported spending more than 40 h online per week, which translates to approximately 5 h a day, and could be interpreted as a pattern of problematic Internet use. According to some authors, there could be a relationship between problematic use and time spent online [29,30]. As expected, the more time spent online, the more problematic use will occur. This may entail consequences that affect important aspects in adolescents; for example, some studies claim that spending a lot of time on the Internet could be related to greater difficulty concentrating on less stimulating tasks, and thus affect academic performance and self-esteem [31,32,33].

No differences were found regarding the use of the Internet by boys and girls, i.e., they use it with the same frequency, although some studies state that boys use ICT for longer periods of time [34,35]. In contrast, other research refers to girls using the Internet more frequently [15,33,36]. One relevant aspect is the type of use by men and women; in this respect, it is believed that they do differ, as women tend to use Internet to interact with their peers, while men use it more for leisure, mostly accessing online games and videos [30].

The results show that both younger and older teens use the Internet with the same frequency [35]. This is an important finding, since as other studies [32,36] show, the time spent using the Internet is greater among teenagers in the second cycle of secondary education. This could be explained by the belief that older adolescents should make more use of the Internet, either for academic reasons or because, in theory, they have a more active social life than 13-year-olds. However, in this study we cannot claim that there is an age or grade difference between teenagers.

Most teenagers spend time on the Internet to keep in touch with their friends. According to other studies [3,37,38], the most common reason for using Internet is to talk to friends through social networks or applications such as WhatsApp. In other words, the main reason for Internet use is entertainment and leisure [39], which is why many individuals do not use the Internet for academic tasks or for personal growth and development. They tend to go online mainly because they feel lonely, because it is easier for them to maintain social relationships online than in person and/or because they enjoy themselves online. This may reflect certain deficits in social skills, low self-esteem or problems adapting to the environment [33]. In any case, further research is needed in this area. By using the Internet for these activities, such issues may be negatively reinforced, resulting in reduced direct social involvement with peers, reduced psychological well-being and maladaptive social construction.

Regarding the use of social networks, WhatsApp, YouTube and Instagram are the most used on a daily basis, and are used most of the time. This shows that they are not a work tool or just another way of communicating, but rather a way of constructing the personality they wish to present, which will depend, on some occasions, on the like responses to the content individuals share with their peers. Teenagers largely use these networks to make themselves visible and present themselves, and the aim of this high exposure is to achieve recognition.

Receiving many likes, and feeling good when receiving them, is related to achieving visibility and fame in networks [29]. In addition, it is also related to self-esteem, since receiving or not receiving recognition is a decision, but to attract attention, it is convenient to have recognition, because not standing out by receiving likes contributes to an individual’s network image, giving them less visibility and a low level of acceptance.

Likewise, fame in networks is influenced by sexist stereotypes through an image shaped by cultural criteria, where the exhibition of personal beauty applies to a greater extent to women, while sporting skills are a more male domain, although not exclusively [27]. Users who achieve popularity on networks possess a series of stereotypical physical attributes to which appearance is added, that is, the construction of the self, in an intentional and voluntary manner [29].

These results should be considered in future research, as psychological theories explaining the use of social networks is often scarce [40], and there is much inconsistency in the analysis regarding the influence of social networks on self-esteem and the impact that they have on users through the responses that users receive [40], such as likes, which undoubtedly affect the construction of their self-esteem [41].

In terms of the limitations of this study, the sample size and type of sampling should be highlighted. For these results to be representative of the province of Huelva, a much larger sample should be studied, including adolescents from secondary schools throughout the province and even from other areas. In addition, another limitation of the study is the social desirability present when answering a questionnaire, especially in teenagers. Future studies should add probabilistic criteria to select their samples, as well as obtaining access to larger samples. In addition, it would be useful to have as homogeneous a sample as possible. Another limitation to highlight for future research is the time period for the collection of information; extending it could better reflect long-term changes. On the other hand, due to the rapid growth of ICT and the creation of new social networks with great frequency, these factors must be considered for the most up-to-date research possible at the time of information collection.

The results only allow us to conclude that the use of social networks and the Internet, in relation to sexist attitudes, influences the self-esteem and social construction of adolescents. Boys present more attitudes related to hostile sexism and negative stereotypes associated with women, although with respect to positive stereotypes or benevolent sexism, both present similar attitudes. It can also be concluded that adolescents make inappropriate and excessive use of social networks, which they cannot do without, generally using them for leisure and contact with their peers to obtain personal benefit or recognition.

In conclusion, further research is needed in this field and more specific guidelines for network use are needed to avoid problematic use. In any case, Internet use in adolescence is quite common according to all the literature consulted; this is almost inevitable, but it should be borne in mind that it seems to normalize with age toward a more professional, less recreational use, and, therefore, toward a use with fewer negative effects. It is important that future research and co-educational interventions also focus on the emergence of benevolent sexist beliefs in adolescence and their relationship with self-esteem and the development of self-concept.

## Figures and Tables

**Table 1:** Response frequencies and percentages.

	Yes (%)	No (%)	Not Sure (%)
I don’t like being with people	12 (3.88)	297 (96.12)	
I like to be in contact with my friends	188 (60.84)	121 (39.16)	
I feel lonely	14 (4.53)	295 (95.47)	
I enjoy it	164 (53.07)	145 (46.93)	
I connect via mobile phone	300 (97.09)	9 (2.91)	
I connect via computer	154 (49.84)	155 (50.16)	
I connect via tablet	100 (32.36)	209 (67.64)	
I connect via another device	65 (21.04)	244 (78.96)	
I have parental controls	17 (5.50)	230 (74.43)	62 (20.06)
I share photos or images on social networks	249 (80.58)	60 (19.42)	
I share videos on social networks	166 (53.90)	142 (46.10)	
I share memes or gifs on social networks	160 (53.90)	149 (48.22)	
When I share, I expect many likes	78 (25.24)	231 (74.76)	
I usually like ideological photos	132 (42.72)	177 (57.28)	
I usually like provocative photos	66 (21.36)	243 (78.64)	
I usually give a like to what I really like	267 (86.41)	42 (13.59)	
The more likes I get, the more satisfied I am	53 (17.15)	221 (71.52)	35 (11.33)

**Table 2:** Mann–Whitney U comparison.

	Self-Esteem	Benevolent Sexism	Hostile Sexism
I don’t like being with people	0.006	0.993	0.809
I like to be in contact with my friends	0.436	0.118	0.049
I feel lonely	0.208	0.174	0.343
I enjoy it	0.041	0.002	0.025
I connect via mobile phone	0.522	0.570	0.638
I connect via computer	0.397	0.753	0.307
I connect via tablet	0.967	0.022	0.004
I connect via another device	0.665	0.030	0.046
I have parental controls	0.969	0.253	0.966
I share photos or images on social networks	0.221	0.009	0.977
I share videos on social networks	0.427	0.266	0.002
I share memes or gifs on social networks	0.227	0.008	0.211
When I share, I expect many likes	0.007	0.000	0.210
I usually like ideological photos	0.815	0.001	0.254
I usually like provocative photos	0.379	0.794	0.017
I usually give a like to what I really like	0.597	0.045	0.260
The more likes I get, the more satisfied I am	0.029	0.001	0.717

## Data Availability

The data presented in this study are available on request from the corresponding author. The data are not publicly available due to their nature.
